# Evaluation of a novel triple-action adulticide containing a pyrethroid, macrocyclic lactone, and fatty acid against pyrethroid-resistant *Aedes aegypti* and *Culex quinquefasciatus (Diptera: Culicidae)*

**DOI:** 10.1093/jme/tjae032

**Published:** 2024-03-09

**Authors:** Keira J Lucas, Rebecca Heinig, Leanne Lake, Katie Williams, Casey Parker-Crockett, Rachel Bales, Decyo McDuffie

**Affiliations:** Collier Mosquito Control District, Naples, FL, USA; Collier Mosquito Control District, Naples, FL, USA; Valent Biosciences, Public Health, Libertyville, IL, USA; Valent Biosciences, Public Health, Libertyville, IL, USA; Azelis, Agricultural and Environmental Solutions, Lake Mary, FL, USA; Collier Mosquito Control District, Naples, FL, USA; Collier Mosquito Control District, Naples, FL, USA

**Keywords:** abamectin, avermectin, fenpropathrin, C8910 fatty acid, organophosphate resistance

## Abstract

Insecticide resistance in mosquito populations has long been recognized as a significant global public health challenge, motivating the development of new control chemistries. ReMoa Tri is a novel triple-action space spray that employs a different mode of action than traditional adult mosquito control formulations. It combines 3 components: fenpropathrin, a mixed-type I/II pyrethroid; abamectin, a macrocyclic lactone; and C8910, a patented fatty acid chain. As an ultra-low volume adulticide, ReMoa Tri has the potential to target mosquito species that are resistant to pyrethroid and organophosphate-based control materials. To determine whether ReMoa Tri effectively targets resistant mosquito species in Florida’s Collier County, United States, we conducted ground-based field cage trials using field-caught pyrethroid-resistant *Culex quinquefasciatus* (Say) and *Aedes aegypti* (L.), of which the latter also displayed developing resistance to organophosphates. Trials were also conducted against the same mosquito populations with Merus 3.0, a pyrethrin-based adulticide used by the Collier Mosquito Control District. ReMoa Tri was effective against Collier’s pyrethroid-resistant *Cx. quinquefasciatus*, resulting in more than 95% mortality in semifield cage trials by 24 h postapplication. Similarly, ReMoa Tri applications against Collier’s pyrethroid-resistant *Ae. aegypti* resulted in 72%–89% mortality at 24 h postapplication and 74%–97% mortality at 48 h postapplication. This study represents the first field data on this novel space spray, and its findings shed light on the performance of ReMoa Tri against local mosquito populations that have developed resistance to currently available adulticides.

## Introduction

Several mosquito-borne viruses transmitted by *Culex quinquefasciatus* (Say), including West Nile virus (WNV) and St. Louis encephalitis, are endemic to the southeastern United States. Furthermore, the southern areas of the United States provide suitable habitats and a high probability for the distribution of the yellow fever mosquito, *Aedes aegypti* (L.) (Monaghan et al. 2019, Khan et al. 2020), which can transmit viruses such as dengue (DENV), chikungunya (CHIKV), yellow fever, and Zika (ZIKV). This is particularly problematic in Florida, where these *Aedes* vectors are prevalent throughout the state (Parker et al. 2019), and vector-borne disease outbreaks are a constant threat, as evidenced by recent local transmission of DENV (Sharp et al. 2019, FLDOH 2023), CHIKV (Kendrick et al. 2014), and ZIKV (Philip et al. 2019).

The most effective method of reducing transmission risk to humans is through integrated mosquito management. Vector control strategies in the region are based primarily on the application of insecticides targeting the juvenile and adult stages (Bowman et al. 2016, McAllister et al. 2020). The Insecticide Resistance Action Committee (IRAC) classifies these insecticides by their mode of action (Sparks et al. 2020). Until recently, only 2 insecticide mode of action classifications have been available for the control of adult mosquitoes in the United States: the voltage-gated sodium channel (VGSC) modulators, known as pyrethroids and pyrethrins (IRAC group 3A), and the acetylcholinesterase inhibitors, known as organophosphates (IRAC group 1B). Resistance to pyrethroid-based adulticides is well documented in Florida for *Ae. aegypti* (McAllister et al. 2020, Estep et al. 2018, Parker et al. 2020, Schluep and Buckner 2021, Scott et al. 2021, Lucas and Bales 2022) and *Cx. quinquefasciatus* (Lucas et al. 2020, McGregor et al. 2021). Organophosphate resistance in Florida has also been detected in *Ae. aegypti*, including those in Collier County (Parker et al. 2020). To prevent and address insecticide resistance, pesticide applicators are advised to engage in product rotation, which involves treating populations with insecticides from different IRAC classes on a revolving basis, e.g., using group 1B organophosphates for a period of time before transitioning to group 3A pyrethroids (Sparks and Nauen 2015). However, only 2 classes of adult mosquito control products (adulticides) have been available in the United States. Thus, domestic mosquito control agencies have traditionally been limited to using either pyrethroids or organophosphates for adult control, making true insecticide rotation challenging, especially when insecticide resistance renders certain pesticide groups ineffective.

Resistance to insecticides in mosquitoes is attributed to several mechanisms, including knockdown (*kdr*) and metabolic resistance. In mosquitoes, *kdr* resistance is linked to point mutations in the VGSC gene. Sodium channels are an essential component of the nerve cell membrane and play a role in nerve-cell excitability. Pyrethroids prolong the opening of sodium channels, causing excitatory paralysis before death occurs, resulting in a “knockdown” effect. Point mutations in the VGSC result in reduced binding of pyrethroids and decreased sensitivity to the insecticide. Consequently, although the knockdown effect is initially observed, insects with these mutations may eventually recover over time (Parker 2020). Another important contributor to resistance is metabolic detoxification (metabolic resistance), where metabolic detoxification enzymes limit an insecticide’s toxic effect by degrading or sequestering the insecticide. This type of resistance is caused by the upregulation of enzymes from large multigene families of oxidases, esterases, and glutathione S-transferases (Brogdon and McAlliser 1998A).

Collier County mosquito populations have displayed resistance to pyrethroid-based products, with many of the mechanisms described above at play (Estep et al. 2018, Lucas et al. 2020, Schluep and Buckner 2021). For *Cx. quinquefasciatus*, *kdr* and metabolic processes play important roles in their pyrethroid-resistant status. The presence of a *kdr* mutation resulting in the conversion of leucine (L) to phenylalanine (F) at position 1014 (L1014F) in the VGSC gene has been identified and shown to contribute to *Cx. quinquefasciatus* resistance status (Lucas et al. 2020). Additionally, detoxification enzyme-related resistance, involving oxidase and esterase activity, is also a significant factor in their resistance levels (Lucas et al. 2020).

Similarly, *Ae. aegypti* mosquitoes in Collier County exhibit high levels of resistance to pyrethroid-based products (Lucas and Bales 2022). These mosquitoes carry a high frequency of *kdr* alleles, including the conversion of valine (V) to isoleucine (I) at position 1016 (V1016I) and F to cysteine (C) at position 1534 (F1534C) (Estep et al. 2018). However, it is not yet confirmed whether these mutations result in resistant phenotypes. Detoxification enzyme-based resistance, particularly related to esterase activity, has also been identified in Collier County *Ae. aegypti* (Schluep and Buckner 2021). Overall, the extensive study of these resistant mosquito strains indicates that multiple factors, including genetic mutations and detoxification enzyme activity, are responsible for the development of pyrethroid resistance in both *Cx. quinquefasciatus* and *Ae. aegypti* populations, rendering their control challenging.

A newly available adulticide for mosquito control, known as ReMoa Tri (4% fenpropathrin; 1.5% abamectin; 1% C8910 FA) (Valent Biosciences, Libertyville, Illinois), is a triple-action adult space spray that combines abamectin (avermectin 1B) with C8910 fatty acid and fenpropathrin. Abamectin (IRAC group 6) is a macrocyclic lactone produced by the soil microorganism *Streptomyces avermitilis* Kim and Goodfellow. Furthermore, C8910 is a food-safe mixture of compounds comprising straight-chain octanoic, nonanoic, and decanoic saturated fatty acids that fall into IRAC group UNE, which encompasses compounds of unknown or uncertain modes of action.

The concept of utilizing abamectin as a space spray to target adult mosquitoes stems from studies showing ivermectin, another member of the avermectin family of compounds, displays insecticidal properties against adult female *Anopheles* mosquitoes when these mosquitoes blood feed on hosts dosed with ivermectin (Chaccour et al. 2010, 2013, Alout et al. 2014). Furthermore, due to abamectin’s success as an agricultural and residential pesticide, the active ingredient was combined with fenpropathrin, a mixed-type I/II pyrethroid, and C8910 FA for use in mosquito control. As an adulticide employing a new mode of action for ultra-low volume (ULV) space spray targeting adult mosquitoes, ReMoa Tri has the potential to successfully control mosquito species resistant to conventional pyrethroid and organophosphate-based products.

To determine whether ReMoa Tri effectively targets local strains of pyrethroid and organophosphate-resistant mosquito species in Collier County, we isolated strains of *Cx. quinquefasciatus* and *Ae. aegypti* from locations that had previously shown evidence of insecticide resistance (Estep et al. 2018, Lucas et al. 2020, Parker et al. 2020, Schluep and Buckner 2021, Lucas and Bales 2022). Each strain’s resistance to common adulticide active ingredients was confirmed in a series of CDC bottle bioassays (Brogdon and McAllister 1998B), which indicated that both strains were resistant to several common pyrethroids and that the *Ae. aegypti* strain was also developing resistance to naled, an organophosphate. We then conducted ground-based field cage trials using ReMoa Tri against resistant local strains and susceptible laboratory strains of each species. For comparison, we also evaluated the efficacy of Merus 3.0 (5% pyrethrins) (Clarke, St. Charles, Illinois), an adulticide used operationally by Collier Mosquito Control District (CMCD), against the same mosquito populations. Here, we report our findings on the effectiveness of ground applications of ReMoa Tri against resistant *Cx. quinquefasciatus* and *Ae. aegypti* under semifield conditions for the first time.

## Materials and Methods

### Mosquito Collections and Rearing

The field strain of *Cx. quinquefasciatus* (Collier-*Cx. quinquefasciatus*) was collected as larvae from a wastewater treatment facility at Big Cypress Elementary School in Collier County (26.22581° N, 81.67200° W). The larvae were separated by species based on morphology, and the *Cx. quinquefasciatus* larvae were brought back to the insectary regulated at 28 °C, 80% relative humidity, and a constant 14-h-light:10-h-dark cycle. Larvae were provided with a diet of equal parts dog chow, lactalbumin, and brewer’s yeast. The resulting adult mosquitoes were reared with a supply of 10% (wt/vol) sucrose solution ad libitum. Susceptible-*Cx. quinquefasciatus* (Benzon Research, Carlisle, Pennsylvania) (susceptible-*Cx. quinquefasciatus*) were received as 3- to 5-day-old adult females and maintained under the conditions described above.

A laboratory strain for *Ae. aegypti* (Collier-*Ae. aegypti*) was developed by placing ovitraps containing seed germination paper and water in Golden Gate City within Collier County (26.178610° N, 81.69421° W). Collected eggs were hatched, and larvae were reared through adult emergence as described above. After identifying adult mosquito species by morphology, *Ae. aegypti* were selected and reared through multiple generations (~12) to establish a laboratory strain. For field cage trials, susceptible Benzon Research 1994 *Ae. aegypti* were received as 3 to 5-day-old adult females and kept in the insectary regulated as described above. Alternatively, for CDC bottle bioassays, susceptible ORL 1952 *Ae. aegypti* were reared in the insectary as described above. Both susceptible strains will be referred to as susceptible*-Ae. aegypti.*

### Pesticide Susceptibility Tests

Three replicates of approximately 15–20 adult female susceptible-*Cx. quinquefasciatus*, Collier-*Cx. quinquefasciatus*, susceptible-*Ae. aegypti*, and Collier-*Ae. aegypti* laboratory strain were exposed to the CDC diagnostic dose of the technical grade materials of either naled (2.25 μg/ml), d-phenothrin (20 μg/ml), or pyrethrum (15 μg/ml). Technical grade products were diluted with acetone to deliver the CDC diagnostic dose of the active ingredient in 1 ml of solution. Wheaton media bottles (250 ml) were coated with one of the test solutions or acetone, which served as the control. Bottles were dried for 2 h in a dark space and used immediately for the assay. Knockdown was recorded at 5, 10, and 15 min, and every 15 min for 2 h thereafter. After 2 h, mosquitoes were transferred from the test bottles to holding cages, and knockdown was recorded at 24 h postexposure. Knockdown was recorded if mosquitoes could no longer stand, displayed erratic behavior, or could not maintain flight. Percent mortality was calculated and corrected using Abbott’s formula (Abbott 1925). Resistance status was defined based on the World Health Organization guidelines (WHO 2013).

### Preparation of Field Equipment and Application Rates

For the field cage trials, 2 ULV formulations were applied at the following label rates: ReMoa Tri was applied at the midlabel rate of 48.89 ml/ha (0.669 oz/acre), and Merus 3.0 was applied at CMCD’s current operational use of 60.65 ml/ha (0.83 oz/acre). ReMoa Tri was diluted in ReMoa diluent (Valent Biosciences, Libertyville, Illinois ) in a 1:2 (diluent:product) mix ratio to achieve the target application rate at a speed of 16.09 kph (10 mph) and a flow of 180.40 ml/min (6.1 oz/min). Merus 3.0 was diluted in Envirotech ULV Diluent Oil (Clarke, St. Charles, Illinois) in a 1:4 (diluent:product) mix ratio to achieve the desirable application rate at the speed above and a flow of 183.36 ml/min (6.2 oz/min). Tinopal OB (BASF, Ludwigshafen, Germany), an ultraviolet dye, was added to each diluted product at a ratio of 2 g/L to facilitate droplet characterization. Each product was applied using a Dyna-Jet L-30 ULV sprayer (Curtis Dyna-Fog Ltd., Jackson, Georgia) outfitted to the back of a Polaris Ranger 1000 (Polaris Inc., Medina, Minnesota). The ULV sprayer was calibrated with each diluted formulated product, and spray droplets were measured using a DC-IV droplet testing system (KLD Labs, Hauppauge, New York) to ensure that they were within each product’s label specifications.

### Field Evaluation of ReMoa Tri and Merus 3.0

The test location was a large open field located at the Collier County Fairgrounds (26.30571° N, 81.58814° W), which provides ample space, no wind obstructions, little human activity, and paved roads, allowing the vehicle to travel in any direction as necessary per wind direction. The field test plot was a 3 × 3 grid design with 3 rows of 3 sampling stations positioned at 30.48 m (100 ft), 60.96 m (200 ft), and 91.44 m (300 ft) downwind from the ULV application line. Sampling station replicates at each position were placed 30.48 m (100 ft) apart perpendicular to the ULV application to create the 3 × 3 grid. Each sampling station included a stand that held one field cage of Collier-strain mosquitoes (Collier-*Cx. quinquefasciatus* or Collier-*Ae. aegypti*) on one side and one field cage of susceptible strain mosquitoes of the same species on the other side. The cages were affixed to a rotating arm with a wind vane to ensure that the cages would be oriented perpendicular to the prevailing wind direction. Each station also included a rotating impinger (Leading Edge, Port Orange, Florida) with 3-mm Teflon-coated acrylic rods to collect the droplets during each application.

Field cage trials for *Cx. quinquefasciatus* occurred on 19 October 2021 using both ReMoa Tri and Merus 3.0. Field cage trials for *Ae. aegypti* occurred on separate days: 14 June 2023 for ReMoa Tri and 14 June 2023 for Merus 3.0. On the day of each trial, ten to twenty 3- to 5-day-old adult female mosquitoes of the appropriate strains were aspirated into each field cage. Each cage was provided with 10% sucrose solution and stored at 28 °C prior to transport to the test location. At the field test plot, 3 control cages of each Collier strain and susceptible strain mosquitoes were hung for 30 min and taken down prior to the start of applications. A weather station tracking humidity, wind speed, wind direction, and temperature at both 9.14 m (30 ft) and 1.5 m (5 ft) from ground level was placed at the application site to monitor atmospheric conditions during the trial. The trials were only conducted during stable atmospheric conditions when wind velocity was 1.6–16 kph (1–10 mph) and a temperature inversion was observed. Treatment parameters for each application are described in Table 1. The drive line was set 30.48 m (100 ft) downwind from the first line of sampling stations. The ULV sprayer was turned on 60.96 m (200 ft) before the first row of sampling stations and turned off 60.96 m (200 ft) after the last row of sampling stations to ensure adequate coverage. Each application was made at 16.09 kph (10 mph) with an anticipated swath of 91.44 m (300 ft).

**Table 1.** Environmental parameters during field cage trials

Date	Time	Product	Temperature (°C)		Wind speed (km/h)	Wind direction	Relative humidity (%)
			1.524 m	9.144 m			
19 October 2021	7:41 PM	Merus 3.0	25.11	25.22	9.98	E	63.3
19 October 2021	8:15 PM	ReMoa Tri	24.22	24.44	5.31	E	67.4
14 June 2023	8:13 PM	ReMoa Tri	30.28	31.11	3.86	W	68.7
15 June 2023	7:53 PM	Merus 3.0	30.67	32.22	6.28	WSW	62.3

The caged mosquitoes and rotating Teflon-coated acrylic rods were collected 20 min postapplication. Upon collection, mosquitoes were immediately aspirated from field cages, transferred to clean holding containers on site, and brought back to the laboratory for evaluation. Mortality was recorded at 24 and 48 h postapplication. Mortality was recorded if mosquitoes could no longer stand, displayed erratic behavior, or could not maintain flight. Corrected percent mortality was calculated using Abbott’s (1925) formula. Graphical analysis and statistical significance using multiple unpaired *t*-tests were determined using GraphPad Prism version 9.0.0 for macOS (GraphPad Software, Boston, Massachusetts). Teflon-coated acrylic rods from each sampling station were analyzed by the A-Drop (Valent Biosciences, Libertyville, Illinois) droplet analysis program.

## Results

### Pesticide Susceptibility Evaluation of Collier-*Cx. quinquefasciatus*

Susceptible-*Cx. quinquefasciatus* and the Collier-*Cx. quinquefasciatus* used in field trials were subjected to CDC bottle bioassays. Three common active ingredients in mosquito adulticide products were chosen for the assay: sumithrin, pyrethrum, and naled. Sumithrin and pyrethrum were chosen because these materials serve as active ingredients in pyrethroid-based control materials used by CMCD. Furthermore, naled is the active ingredient of an organophosphate-based insecticide, Dibrom Concentrate (AMVAC, Newport Beach, California), also used by CMCD.

**Fig. 1.**
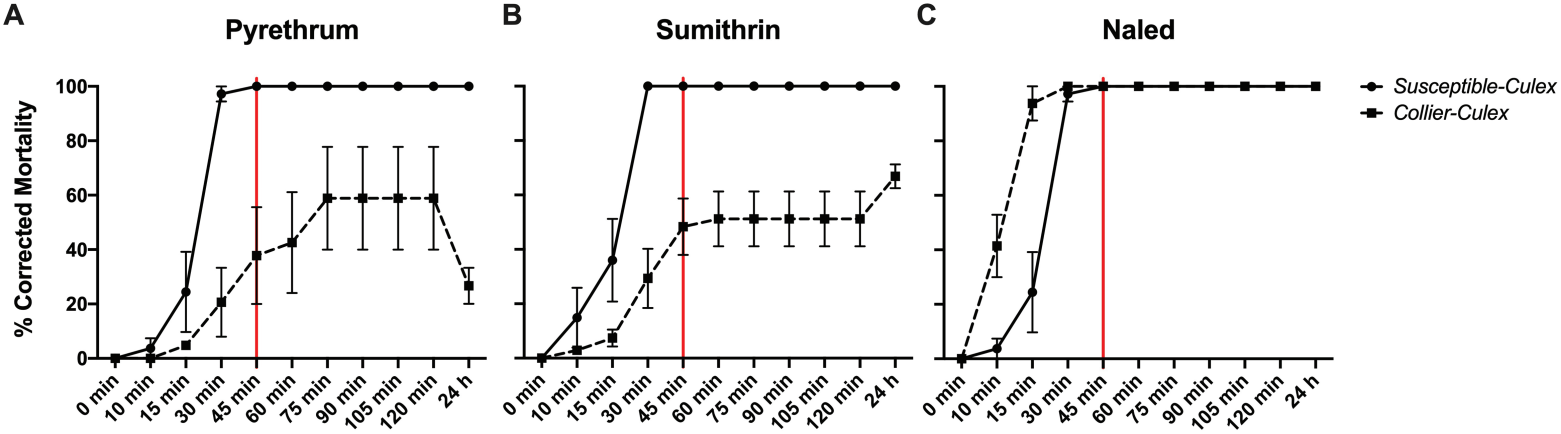
Centers for Disease Control and Prevention (CDC) bottle bioassays for susceptible-*Culex quinquefasciatus* (solid line) and Collier-*Cx. quinquefasciatus* (dashed line). A–C) CDC bottle bioassays using technical grade insecticides: A) 15 μg/ml pyrethrum, B) 22 μg/ml d-phenothrin (Sumithrin) and C) 2.25 μg/ml naled. A solid vertical red line indicates the published threshold for the CDC diagnostic dose of the susceptible-*Cx. quinquefasciatus* Sebring colony. Data represent 3 technical replicates and are shown as mean ± SEM.

As expected, susceptible-*Cx. quinquefasciatus* displayed 100% mortality occurring at the CDC diagnostic time of 45 min for all 3 technical grade materials tested. The Collier-*Cx. quinquefasciatus* exhibited resistance to pyrethroids, with 37.78 ± 17.78% knockdown for pyrethrum (Fig. 1A) and 48.33 ± 10.35% knockdown for sumithrin (Fig. 1B) at the diagnostic time. Even after 2 h of exposure, knockdown remained low at 58.89 ± 18.89% and 51.23 ± 10.071% for pyrethrum and sumithrin, respectively. Collier-*Cx. quinquefasciatus* was susceptible to the organophosphate naled, with a complete knockdown at diagnostic time (Fig. 1C). At 24 h postexposure with pyrethrum, Collier-*Cx. quinquefasciatus* knockdown decreased from 58.89 ± 18.89% to 26.67 ± 6.67% (Fig. 1A), a 32% recovery, indicating possible *kdr*-associated resistance. Taken together, these data indicate that the Collier-*Cx. quinquefasciatus* strains used in the field trials are resistant to pyrethroid-based insecticides but susceptible to organophosphate-based materials.

### Pesticide Susceptibility Evaluation of Collier-*Ae. aegypti*

**Fig. 2.**
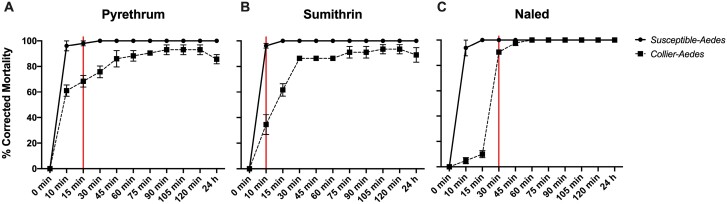
Centers for Disease Control and Prevention (CDC) bottle bioassays for susceptible-*Ae. aegypti* (solid line) and Collier-*Ae. aegypti* (dashed line). A–C) CDC bottle bioassays using technical grade insecticides: A) 15 μg/ml pyrethrum, B) 22 μg/ml d-phenothrin (Sumithrin), and C) 2.25 μg/ml naled. A solid vertical red line indicates the published threshold for CDC diagnostic dose of the susceptible-*Ae. aegypti* REX colony. Data represent 3 technical replicates and are shown as mean ± SEM.

Collier-*Ae. aegypti* used in field trials and susceptible-*Ae. aegypti* (ORL 1952) were subjected to CDC bottle bioassay as described above. As expected, susceptible-*Ae. aegypti* displayed 100% mortality at the CDC diagnostic time for the pyrethroid-based technical grade products and nearly 100% mortality for technical grade naled (Fig. 2). The Collier-*Ae. aegypti* strain exhibited resistance to pyrethroids, with 68.38 ± 7.83% mortality at 15 min for pyrethrum (Fig. 2A) and 34.52 ± 13.52% mortality at 10 min for sumithrin (Fig. 2B). After 2 h of exposure, mortality reached 92.99 ± 6.69% and 93.45 ± 6.27% for pyrethrum and sumithrin, respectively. Collier-*Ae. aegypti* displayed “developing resistance” to the organophosphate naled, with 90.43 ± 3.26% knockdown at the CDC diagnostic time of 30 min for naled (Fig. 2C). Furthermore, a 4.46%–7.35% recovery for Collier-*Ae. aegypti* was observed at 24 h postexposure for both sumithrin and pyrethrum (Fig. 2A and B), indicating possible *kdr*-associated resistance. These data signify that Collier-*Ae. aegypti* used in the field trials are resistant to pyrethroid-based insecticides and developing resistance to naled.

### Applications of ReMoa Tri and Merus 3.0 Targeting Collier-*Cx. quinquefasciatus*

**Fig. 3.**
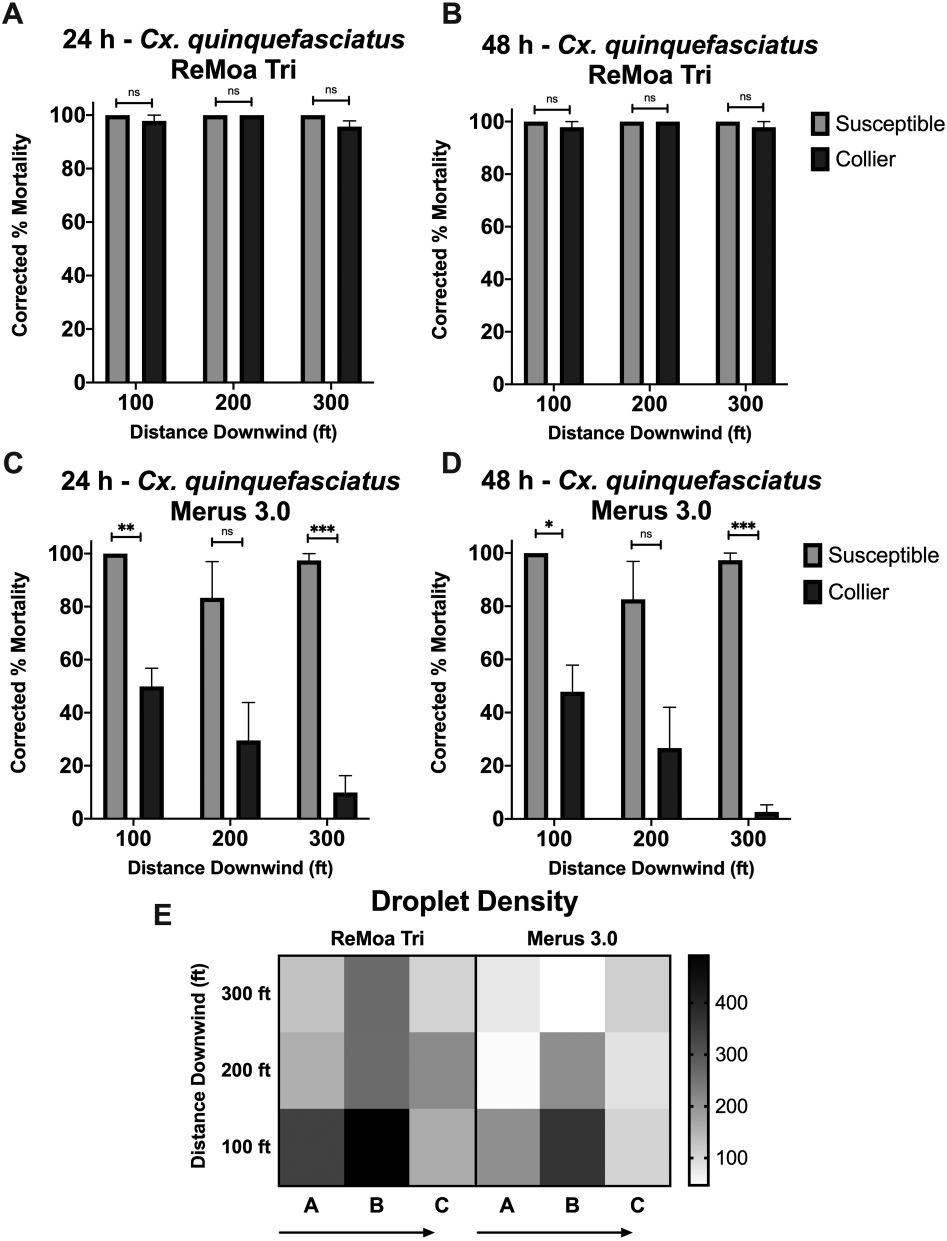
Ground-based field cage trials using ReMoa Tri and Merus 3.0 against susceptible-*Cx. quinquefasciatus* and Collier-*Cx. quinquefasciatus*. A and B) *Cx. quinquefasciatus* mortality at 24 h A) and 49 h B) postapplication of ReMoa Tri. C and D) *Cx. quinquefasciatus* mortality at 24 h C) and 49 h D) postapplication of Merus 3.0. A–D) Data represent 3 technical replicates and are shown as mean ± SEM. Multiple unpaired *t*-tests were performed to indicate statistical significance: ns = not significant; *P < 0.05; **P < 0.01; ***P < 0.001. E) Droplet density (mm^2^) for ReMoa Tri (left) and Merus 3.0 (right) applications at each sampling station. Arrows indicate the direction of the driveline.

ReMoa Tri was effective against the susceptible-*Cx. quinquefasciatus*, with 100% mortality at each distance downwind of the spray origin at 24 h (Fig. 3A) and 48 h (Fig. 3B) postapplication. By 24 h and continuing through 48 h postapplication, the Collier-*Cx. quinquefasciatus* reached 97.9 ± 2.1% (average corrected percent mortality ± SEM), 100% and 95.7 ± 2.1% mortality at 30.48, 60.96, and 91.44 m, respectively (Fig. 3A and B). No significant difference in effectiveness was observed between the susceptible-*Cx. quinquefasciatus* and the Collier-*Cx. quinquefasciatus*, indicating that ReMoa Tri was just as effective at knocking down pyrethroid-resistant *Cx. quinquefasciatus* as susceptible-*Cx. quinquefasciatus.* Furthermore, no recovery in mortality was observed between 24 h and 48 h postapplication in the Collier-*Cx. quinquefasciatus.* Nontreated control susceptible-*Cx. quinquefasciatus* displayed no mortality at 24 h postapplication, and 4.7 ± 2.5% mortality at 48 h postapplication, while nontreated Collier-*Cx. quinquefasciatus* displayed 2.8 ± 2.7% mortality at 24 h and 48 h postapplication. Together, these results indicate ReMoa Tri is an effective space spray for targeting both susceptible and pyrethroid-resistant *Cx. quinquefasciatus.*

Merus 3.0 was also effective against the susceptible-*Cx. quinquefasciatus*, with 100%, 83.33 ± 13.66%, and 97.44 ± 2.56% mortality at 30.48, 60.96, and 91.44 m, respectively, at 24 h postapplication (Fig. 3C and D). Furthermore, reduced effectiveness was observed for Merus 3.0 with the Collier-*Cx. quinquefasciatus*, with 49.90 ± 6.89%, 29.54 ± 14.32%, and 9.90 ± 6.35% mortality at 30.48, 60.96, and 91.44 m, respectively, at 24 h postapplication (Fig. 3C and D). Mortality also varied across the application swath. Collier-*Cx. quinquefasciatus* displayed significantly reduced mortality compared to susceptible-*Cx. quinquefasciatus* at the 30.48 m (*P* = 0.0038, df = 4) and 91.44 m (*P* = 0.0006, df = 4) sampling stations at 24 h postapplication. A similar trend was observed for the 48 h postapplication time point with Collier-*Cx. quinquefasciatus* displaying significantly reduced mortality compared to susceptible-*Cx. quinquefasciatus* at the 30.48 m (*P* = 0.0130, df = 4) and 91.44 m (*P* = 0.00004, df = 4) sampling stations at 48 h postapplication (Fig. 3C and D). Furthermore, recovery in mortality was observed in the 100 A, 100 C, 200 C, and 300 C cages for the Collier-*Cx. quinquefasciatus*. Nontreated control susceptible-*Cx. quinquefasciatus* displayed no mortality at 24 h postapplication, and 4.1 ± 2.5% mortality at 48 h postapplication, while nontreated Collier-*Cx. quinquefasciatus* displayed 4.2 ± 2.7% mortality at 24 h and 48 h postapplication. The reduced mortality in the susceptible strain seen at the 60.96 m sampling stations is likely due to the reduced droplet density in the A and C stations (Fig. 3E). These results suggest that Merus 3.0 is an effective space spray when targeting susceptible-*Cx. quinquefasciatus* but not effective in targeting pyrethroid-resistant *Cx. quinquefasciatus*.

### Applications of ReMoa Tri and Merus 3.0 Targeting Collier-*Ae. aegypti*

**Fig. 4.**
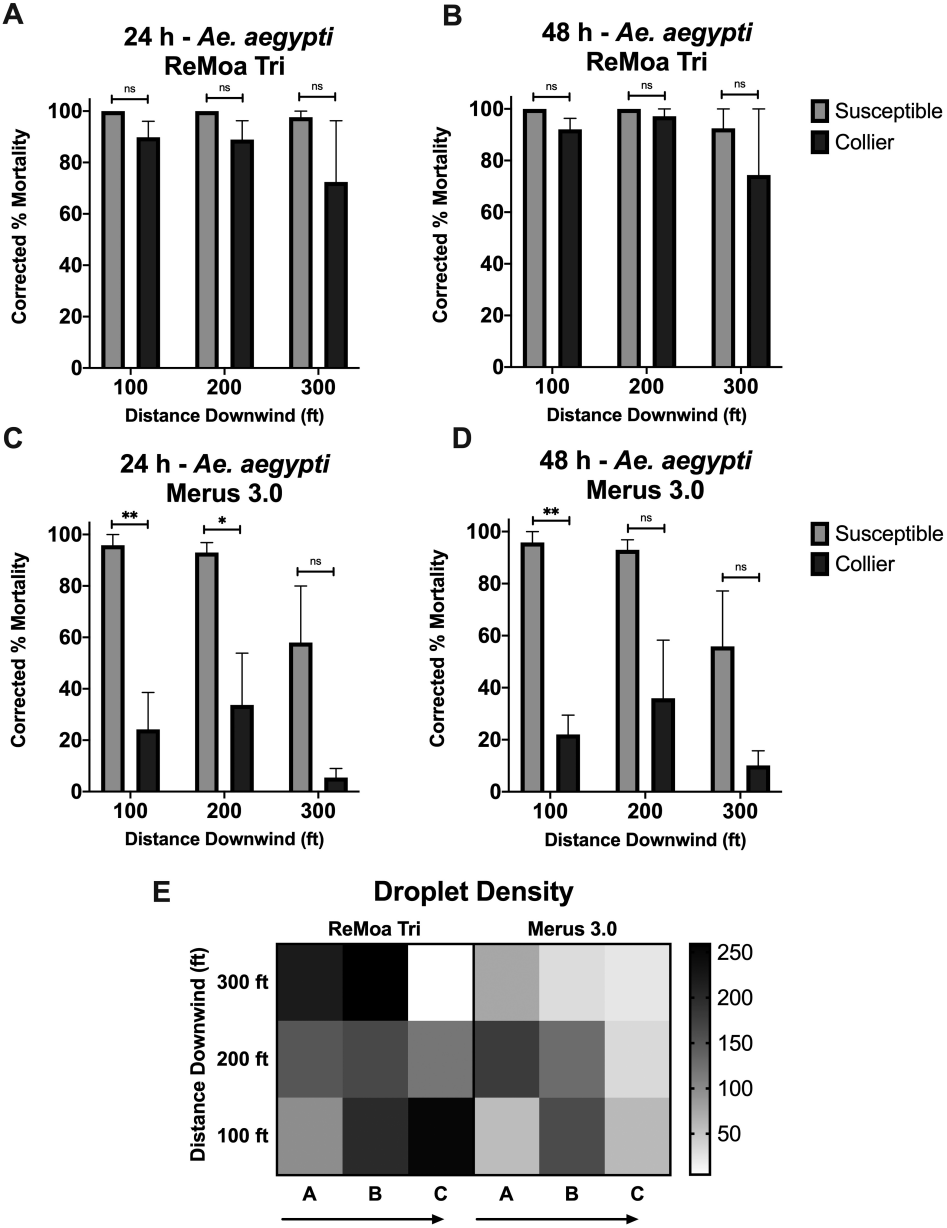
Ground-based field cage trials using ReMoa Tri and Merus 3.0 against susceptible-*Ae. aegypti* and Collier-*Ae. aegypti*. A–B) *Ae. aegypti* mortality at 24 h A) and 49 h B) postapplication of ReMoa Tri. C and D) *Ae. aegypti* mortality at 24 h C) and 49 h D) postapplication of Merus 3.0. A–D) Data represent 3 technical replicates and are shown as mean ± SEM. Multiple unpaired *t*-tests were performed to indicate statistical significance: ns = not significant; *P < 0.05; **P < 0.01. E) Droplet density (mm^2^) for ReMoa Tri (left) and Merus 3.0 (right) applications at each sampling station. Arrows indicate the direction of the driveline.

As was the case in the field cage trials for *Cx. quinquefasciatus*, ReMoa Tri was effective against the susceptible-*Ae. aegypti*. At 24 and 48 h postapplication, the susceptible-*Ae. aegypti* reached 100% mortality at the 30.48- and 60.96-m distances and 97.62 ± 2.38% mortality at the 91.44 m distance downwind of the spray origin (Fig. 4A). By 24 h postapplication, the Collier-*Ae. aegypti* reached 89.82 ± 6.21%, 88.89 ± 7.35%, and 72.43 ± 23.82% mortality at 30.48 m, 60.96 m, and 91.44 m, respectively (Fig. 4A and B). And by 48 h postapplication, the Collier-*Ae. aegypti* reached 92.09 ± 4.27%, 97.15 ± 2.85%, and 74.39 ± 25.61% mortality at 30.48, 60.96, and 91.44 m, respectively (Fig. 4A and B). The reduced mortality in the susceptible-*Ae. aegypti* and Collier-*Ae. aegypti* at 91.44 m is likely due to the reduced droplet density at the 300 C station (Fig. 4E). Nontreated control susceptible-*Ae. aegypti* displayed no mortality at 24 h postapplication and 5.1 ± 5.1% mortality at 48 h postapplication. Nontreated Collier-*Ae. aegypti* displayed no mortality at 24 h postapplication and 2.9 ± 2.4% mortality at 48 h postapplication. No significant difference in effectiveness was observed between the susceptible-*Ae. aegypti* and the Collier-*Ae. aegypti,* indicating ReMoa Tri was just as effective at knocking down pyrethroid-resistant *Ae. aegypti* with developing resistance (per WHO guidelines) to naled as susceptible-*Ae. aegypti.* Furthermore, no recovery in mortality was observed between 24 and 48 h postapplication in the Collier-*Ae. aegypti.* Together, these results indicate ReMoa Tri is an effective space spray for targeting susceptible and pyrethroid and naled-developing resistance *Ae. aegypti.*

Merus 3.0 was also effective against the susceptible-*Ae. aegypti*, with 95.83 ± 4.18%, 93.0 ± 3.86%, and 57.97 ± 22.02% mortality at 30.48, 60.96, and 91.44 m, respectively, at both 24 and 48 h postapplication (Fig. 4C and D). By 24 h postapplication, the Collier-*Ae. aegypti* had 24.16 ± 14.36%, 33.64 ± 20.16%, and 5.41 ± 3.52% mortality at 30.48, 60.96, and 91.44 m, respectively (Fig. 4C and D). And by 48 h postapplication, the Collier-*Ae. aegypti* reached 22.01 ± 7.46%, 35.92 ± 22.37%, and 10.14 ± 5.61% mortality at 30.48, 60.96, and 91.44 m, respectively (Fig. 4C and D). The Collier-*Ae. aegypti* displayed significantly reduced mortality compared to susceptible-*Ae. aegypti* at the 30.48 m (*P* = 0.0087, *df* = 4) and 60.96 m (*P* = 0.0445, *df* = 4) sampling stations at 24 h postapplication, and the 30.48 m (*P* = 0.0010, *df* = 4) at 48 h postapplication. Nontreated control susceptible-*Ae. aegypti* displayed no mortality at 24 or 48 h postapplication. Nontreated Collier-*Ae. aegypti* displayed 2.6 ± 2.6% mortality at 24 h and 48 h postapplication. The reduced mortality in the susceptible strain seen at the 91.44-m sampling station is likely due to the reduced droplet density in the A and C stations (Fig. 4E). These results suggest that Merus 3.0 is an effective space spray when targeting susceptible-*Ae. aegypti* but not effective in targeting pyrethroid-resistant *Ae. aegypti* with developing resistance to naled.

## Discussion

The issue of insecticide resistance in mosquito populations poses a substantial global public health concern, especially in areas where mosquito-borne diseases such as malaria, DENV, ZIKV, and WNV are widespread (Nauen 2007). It is, therefore, crucial to assess resistance status and select control materials that are effective against resistant species. In Collier County, this issue is acutely manifested, with documented resistance to pyrethroids in *Cx. quinquefasciatus* (Lucas et al. 2020) and to both pyrethroids and organophosphates in *Ae. aegypti* (Estep et al. 2018, Parker et al. 2020, Schluep and Buckner 2021, Lucas and Bales 2022). Unfortunately, the lack of available options for adulticide product rotations in mosquito control contributes to the heightened complexity of combating resistance and achieving effective control of adult populations.

As a triple-action space spray, ReMoa Tri offers an alternative to traditional pyrethroid and organophosphate-based control materials and has the potential to successfully target resistant mosquito populations. Ground-based applications of ReMoa Tri were performed against pyrethroid-resistant *Cx. quinquefasciatus* and *Ae. aegypti*, of which the latter is also developing resistance to organophosphates in field cage trials. Applications of ReMoa Tri were effective against both susceptible and pyrethroid-resistant *Cx. quinquefasciatus*. Similarly, ReMoa Tri was effective against both susceptible and resistant *Ae. aegypti*. As expected, when resistant *Cx. quinquefasciatus* and *Ae. aegypti* were challenged with Merus 3.0, a pyrethrin-based product, and reduced effectiveness was observed.

Despite its pyrethroid component, ReMoa Tri is specifically labeled for operational control of permethrin-resistant mosquitoes, and our study is the first to provide substantiating evidence in support of this designation. Furthermore, our study highlights the ability of ReMoa Tri to effectively target insecticide-resistant mosquitoes with both single (Lucas et al. 2020) and double (Estep et al. 2018) *kdr* mutations as well as metabolic-associated resistance attributed to oxidase and esterase activity (Lucas et al. 2020, Schluep and Buckner 2021). The efficacy of ReMoa Tri can likely be attributed to its triple-action insecticidal formulation, which capitalizes on 3 distinct modes of action: (i) fenpropathrin, a mixed-type I/II pyrethroid that targets the mosquito VGSC (Gammon et al. 1981, Sparks and Nauen 2015), (ii) abamectin, a macrocy,clic lactone that functions as a glutamate-gated chloride channel allosteric modulator (Sparks and Nauen 2015), and (iii) C8910 FA, which results in respiratory inhibition (Reifenrath 2010) and increased penetrance of the insecticide (Ramadan et al. 2022).

Furthermore, cross-resistance, a phenomenon where mosquitoes exhibit resistance to multiple active ingredients through a unified mechanism, has also been documented in multiple locations (Bisset et al. 1997, Brengues et al. 2003, Ramkumar and Shivakumar 2015, Moyes et al. 2021). This was reflected in our CDC bottle bioassay results, which indicated resistance to two distinct pyrethroid active ingredients. While CMCD has a limited history of pyrethroid use for mosquito control, resistance due to the use of dichlorodiphenyltrichloroethane (DDT) for mosquito control or commercial and residential use of pyrethroid-based materials is a possible explanation. Products relying solely on a single pyrethroid active ingredient might encounter challenges with mosquitoes that have developed cross-resistance mechanisms; however, by incorporating diverse modes of action, products like ReMoa Tri can potentially circumvent these cross-resistance mechanisms. This is evident in our study, where the combined effects of abamectin and fatty acid proved lethal against resistant mosquito populations in Collier County. Such a multifaceted approach, referred to as “multiple attack” in insecticide resistance management (Tabashnik 1989), likely plays a crucial role in the effectiveness of ReMoa Tri against the tested mosquito populations. Conversely, Merus 3.0, which contains pyrethrins (group 3A) exclusively, was not able to overcome the resistance mechanisms that were indicated in the CDC bottle bioassay data and previous studies. In a similar field cage trial in California, mortality rates in local *Cx. quinquefasciatus* were lower compared to a susceptible strain when exposed to Merus 3.0 (Hung et al. 2021), echoing our study’s findings. Similarly, previous field cage trials in Collier County have indicated reduced mortality rates in local *Ae. aegypti* compared to susceptible strains when treated with several pyrethroid-based products, including Merus 3.0 (Lucas and Bales 2022).

This report highlights ReMoa Tri as a practical solution for managing resistant mosquito populations, thereby enhancing the effectiveness and sustainability of mosquito control activities. The documented success of ReMoa Tri in combating both susceptible and resistant mosquito species, as evidenced in this study, underscores its potential to safeguard public health and revitalize the efficacy of mosquito control. Despite ReMoa Tri’s specific labeling for resistant mosquito species, the importance of chemical rotations remains paramount to prevent resistance development or further drive baseline resistance to any of its 3 active ingredients. Resistance to avermectins has been documented in several agricultural pests (Clark et al. 1995), suggesting that the use of abamectin for mosquito control should be used responsibly and in conjunction with other integrated mosquito management methods. Furthermore, ReMoa Tri’s triple-action formulation masks pyrethroid resistance, and its pyrethroid component, fenpropathrin, may continue to drive this resistance in mosquito populations if the product is misused. Notwithstanding these concerns, ReMoa Tri occupies a crucial niche within integrated mosquito management programs and, when used on a rotational basis, will assist in mitigating the selective pressures that have historically fueled resistance development against conventional adulticides.
